# Differential Induction of Resistance Mechanisms by Methyl Jasmonate in Two *Vaccinium corymbosum* L. Cultivars Under Combined Water Deficit and Aluminum Toxicity

**DOI:** 10.3390/plants14203202

**Published:** 2025-10-18

**Authors:** Cristina Cáceres, Crystal Cazor-Curilef, Patricio Delgado-Santibañez, Jorge González-Villagra, Paz Cárcamo-Fincheira, Mabel Delgado, Alejandra Ribera-Fonseca, Claudio Inostroza-Blancheteau, Leon A. Bravo, Adriano Nunes-Nesi, Marjorie Reyes-Díaz

**Affiliations:** 1 Programa de Doctorado en Ciencias de Recursos Naturales, Universidad de La Frontera, Temuco 4811230, Chile; c.caceres02@ufromail.cl; 2 Laboratorio de Ecofisiología Molecular y Funcional de Plantas, Departamento de Ciencias Químicas y Recursos Naturales, Facultad de Ingeniería y Ciencias, Universidad de La Frontera, Temuco 4811230, Chile; 3 Carrera de Bioquímica, Facultad de Ingeniería y Ciencias, Universidad de La Frontera, Temuco 4811230, Chile; c.cazor01@ufromail.cl (C.C.-C.); p.delgado08@ufromail.cl (P.D.-S.); 4 Escuela de Agronomía, Facultad de Ciencias, Ingeniería y Tecnología, Universidad Mayor, Temuco 4780000, Chile; jorge.gonzalez@umayor.cl; 5 Escuela de Agronomía, Facultad de Medicina Veterinaria y Agronomía, Universidad de Las Américas, Santiago 7500975, Chile; mcarcamo@udla.cl; 6 Center of Plant, Soil Interaction and Natural Resources Biotechnology, Scientific and Technological Bioresource Nucleus (BIOREN-UFRO), Universidad de La Frontera, Temuco 4811230, Chile; mabel.delgado@ufrontera.cl (M.D.); alejandra.ribera@ufrontera.cl (A.R.-F.); leon.bravo@ufrontera.cl (L.A.B.); 7 Centro de Fruticultura, Facultad de Ciencias Agropecuarias y Forestales, Universidad de La Frontera, Temuco 4811230, Chile; 8 Laboratorio de Fisiología y Biotecnología Vegetal, Departamento de Ciencias Agropecuarias y Acuícolas, Facultad de Recursos Naturales, Universidad Católica de Temuco, Temuco 4781312, Chile; claudio.inostroza@uct.cl; 9 Núcleo de Investigación en Producción Alimentaria, Facultad de Recursos Naturales, Universidad Católica de Temuco, Temuco 4781312, Chile; 10 Agencia Nacional de Investigación y Desarrollo-Millennium Nucleus in Data Science for Plant Resilience (Phytolearning), Santiago 8370186, Chile; 11 Laboratorio de Fisiología y Biología Molecular Vegetal, Departamento de Ciencias Agronómicas y Recursos Naturales, Facultad de Ciencias Agropecuarias y Medioambiente, Universidad de La Frontera, Temuco 4811230, Chile; 12 National Institute of Science and Technology on Plant Physiology Under Stress Conditions, Departamento de Biologia Vegetal, Universidade Federal de Viçosa, Viçosa 36570-900, MG, Brazil; nunesnesi@ufv.br; 13 Water Research Center for Agriculture and Mining—CRHIAM, Anid Fondap Center, Victoria 1295, Concepción 4070411, Chile

**Keywords:** auxins, drought resistance, highbush blueberry, gene expression, osmolytes, phytohormones

## Abstract

This study aimed to determine the stress mechanisms induced by foliar methyl jasmonate (MeJA) application in *Vaccinium corymbosum* cultivars subjected to water deficit (WD) and aluminum toxicity (Al). Two *V. corymbosum* cultivars, Star and Legacy, were subjected to different treatments in an Andisol: control (80% field capacity and low Al saturation), combined WD + Al (50% field capacity and 85% Al saturation), and different concentrations of foliar MeJA application (10 μM, 50 μM, and 100 μM) under WD + Al conditions. The determination of photosynthetic pigments, osmolytes, and organic acids, as well as the auxin levels and the expression of *Aluminium-Activated Malate Transporter* (*ALMT*) and *Multidrug and Toxic Compound Extrusion* (*MATE*) genes, was analyzed at 7 and 21 days. Foliar MeJA application increased chlorophyll a, b, and carotenoid levels, mainly at 50 µM, exhibiting early Star responses with up to 1.5-fold higher pigment accumulation, and a later increase in Legacy with up to 1.4-fold higher accumulation. Proline increases up to 2.2-fold in roots and sugar by 1.4-fold in leaves of both cultivars. The MeJA application increases the auxin levels by up to 2.3-fold in Star roots at 7 days and by up to 1.4-fold in Legacy leaves at 21 days. MeJA-induced upregulation of *ALMT* and *MATE* gene expression facilitated Al detoxification, with malate and citrate levels increasing up to 2-fold. Hierarchical clustering confirmed that the Star cultivar activated resistance mechanisms early, while the Legacy cultivar exhibited delayed but sustained resistance mechanisms. MeJA improves *V. corymbosum* resistance to combined WD + Al stress by modulating photosynthetic pigments, osmolytes, organic acids, and hormone regulation. This finding underscores the biotechnological potential of MeJA application to improve stress resilience and optimize crop performance under adverse environmental conditions.

## 1. Introduction

Approximately 40% of the land surface is covered by acid soils, which correspond to 70% of the potentially arable land. However, these soils present severe limitations due to aluminum toxicity and water scarcity. The combination of water deficit and aluminum toxicity (WD + Al) exerts considerable stress on plants, significantly affecting crop growth [1,2,3,4]. The highbush blueberry (*Vaccinium corymbosum* L.), introduced in Chile in the 1980s, is a key fruit shrub for the national economy; however, it is exposed to both water deficit and Al toxicity [5,6]. Cáceres et al. [7] demonstrated that plants of highbush blueberry exposed to WD + Al stress exhibited an increase in lipid peroxidation and Al accumulation, decreasing water potential (Ψw), relative water content (RWC), relative growth rate (RGR), and a reduction in net photosynthesis (Pn), stomatal conductance (gs) and transpiration rate (E), compared to plants without combined WD + Al stress. In the same study, two *V. corymbosum* cultivars were identified with contrasting responses to combined WD + Al stress. The Star cultivar demonstrated sensitivity, while the Legacy cultivar exhibited a resistant response.

On the other hand, the use of methyl jasmonate (MeJA) application, a phytohormone that plays a pivotal role in the induction of stress resistance mechanisms, to alleviate the damage caused by WD + Al stress and by individual WD and Al stresses has been proposed as a viable strategy [6,7,8]. MeJA is biosynthesized from linoleic acid, and its mechanism of action involves activating transcription factors that regulate genes involved in abiotic stress resistance. Consequently, it can regulate the accumulation of bioactive compounds and endogenous hormone levels [9,10,11].

Studies have indicated that MeJA stimulates the accumulation of photosynthetic pigments and osmolytes in plants subjected to individual WD or Al stress [12,13]. It has been observed that under WD or Al stress, MeJA increases the levels of photosynthetic pigments such as chlorophylls and carotenoids [6,12]. Higher levels of chlorophylls, mainly Chl b, improve photosynthetic efficiency, as these pigments favor the formation of Chl a/b, increasing the size of light-harvesting complexes (LHCs) antennae [14]. In turn, carotenoids play a key role in dissipating excess light energy through non-photochemical quenching (NPQ) [15]. Under WD conditions, MeJA has been shown to promote the accumulation of osmolytes, including proline and soluble carbohydrates, in species such as *Brassica juncea*, *Ocimum basilicum*, *Glycine max*, and *Fragaria x ananassa* [13,16,17,18]. These osmoregulatory compounds play an essential role in osmotic adjustment in plants. They help maintain an osmotic balance between the inside and outside of cells, allowing plant cells to retain water and maintain turgor pressure, preventing dehydration [19].

Foliar application of MeJA under individual WD and Al stresses can also induce the accumulation and molecular regulation of organic acids (OAs) transport [16,20,21]. OAs play a crucial role in the process of intracellular detoxification of Al through a combination of chelation and vacuolar sequestration. This prevents Al from damaging structures such as cell walls and plasma membranes while improving water and nutrient uptake [22,23]. In the context of molecular regulation, MeJA has been demonstrated to induce the expression of genes associated with organic acid exudation [20,21]. For example, in *Solanum lycopersicum*, foliar application of MeJA increases the expression of the *ALMT* gene, which encodes a malate transporter protein involved in Al tolerance [21]. Similarly, in *Nicotiana tabacum*, MeJA induces the expression of *MATE* family genes responsible for encoding citrate transporter proteins, which are also associated with Al detoxification [20].

Foliar application of MeJA has also been observed to play a key role in regulating endogenous phytohormone signaling networks, promoting the accumulation of hormones such as auxins (AUXs), in *Malus hupehensisse*, *Zea mays*, and *Arabidopsis* mutants [24,25,26]. AUXs regulate plant growth and development and induce the transport of OAs in roots, facilitating the neutralization of toxic metals such as Al [27]. In *Triticum aestivum* L. plants under Al stress, an observation was made that the increase in *ALMT* expression correlates directly with increases in AUXs content. It is proposed that AUXs could be involved in the modulation of mitogen-activated protein kinases (MAPK), which regulate the opening of ALMT anion channels, thereby facilitating malate transport [28].

This study aimed to test the hypothesis that the foliar MeJA application would differentially activate resistance mechanisms in *V. corymbosum* cultivars subjected to combined WD and Al stresses. The investigation focused on changes in photosynthetic pigment content, osmolyte accumulation, organic acid levels, hormone regulation mediated by AUXs, and *ALMT* and *MATE* gene expressions.

## 2. Results

### 2.1. Effect of MeJA Foliar Application on Photosynthetic Pigment Levels

**Figure 1:**
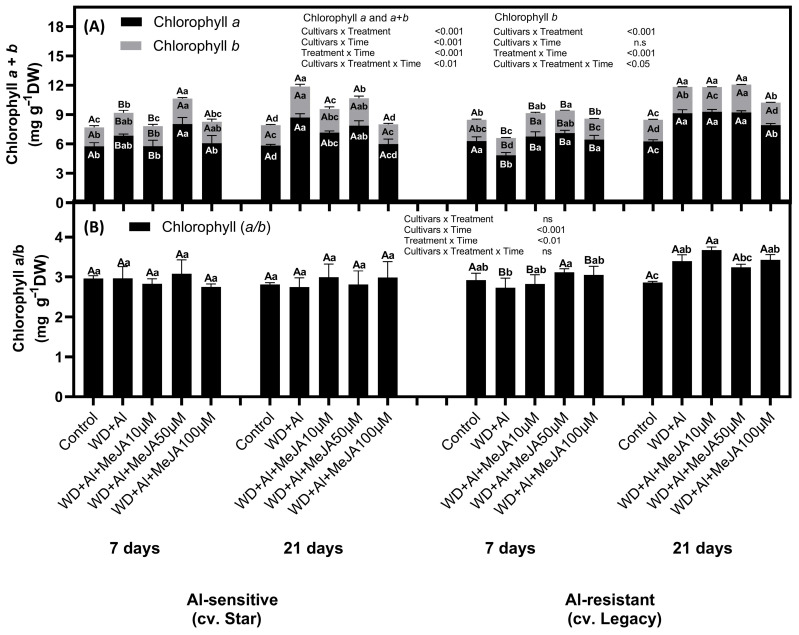
Chlorophyll pigments in *V. corymbosum* cultivars differing in sensitivity to combined water deficit and aluminum stress (WD + Al): sensitive (Star) and resistant (Legacy). Plants were exposed to WD + Al conditions and treated with foliar applications of methyl jasmonate (MeJA) at concentrations of 10, 50, and 100 µM. Panel (**A**) displays the quantification of chlorophyll a, b, and total chlorophyll (a + b), while panel (**B**) shows the chlorophyll a/b ratio. Data correspond to the mean ± standard deviation (SD) of five biological replicates per treatment. Distinct lowercase letters indicate significant differences (Tukey’s test, *p* ≤ 0.05) between treatments within the same cultivar and sampling time; and capital letters indicate significant differences (Tukey’s test, *p* ≤ 0.05) between sampling times within the same treatment and cultivar.

Under WD + Al treatment, the WD + Al-sensitive cultivar (Star) showed significant increases in chlorophyll a, b, and a + b levels compared to control plants, reaching 1.2-fold higher at 7 days and 1.5-fold higher at 21 days of treatment (Figure 1). In contrast, the WD + Al-resistant cultivar (Legacy) showed a significant decrease in chlorophyll a, b, and a + b levels, decreasing up to 0.7-fold compared to control plants at 7 days. However, at 21 days, these levels increased significantly, reaching levels 1.4 times higher than the control plants. As for foliar MeJA application, in the WD + Al-sensitive cultivar, the 50 µM dose led to a significant increase in chlorophyll a, b, and a + b levels at 7 days, reaching higher values than those observed in plants subjected to combined WD + Al stress. In the WD + Al-resistant cultivar, all MeJA doses at 7 days mitigated the decrease in chlorophyll caused by the combined stress, achieving levels statistically comparable to those of control plants. At 21 days, all MeJA doses increased chlorophyll a concentration compared to control plants, reaching levels similar to those observed in plants treated with WD + Al without MeJA. The chlorophyll a/b ratio in the WD + Al-sensitive cultivar did not show consistent differences at 7 or 21 days of treatment among the different treatments. In the WD + Al-resistant cultivar, at 21 days, a substantial increase in the chlorophyll a/b ratio was observed in both the WD + Al without MeJA treatment and in the MeJA treatments at all doses, compared to control plants.

**Figure 2:**
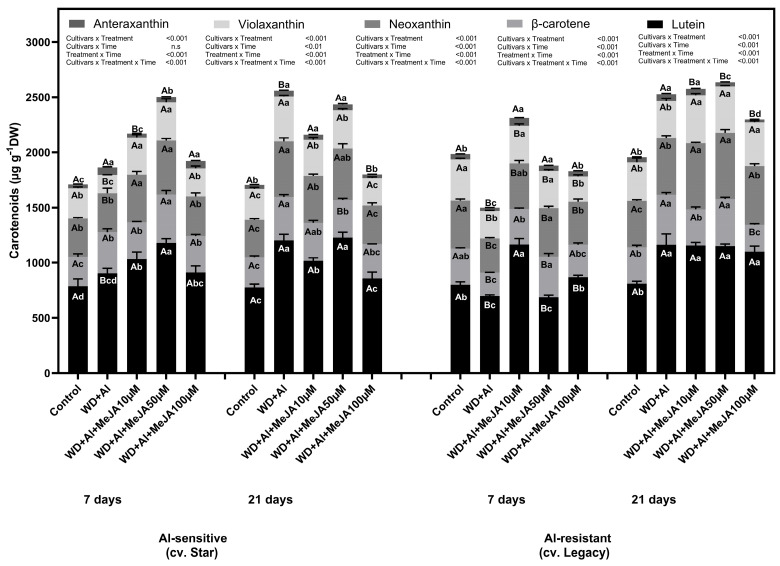
Carotenoid accumulation in two *V. corymbosum* cultivars with contrasting tolerance to combined water deficit and aluminum toxicity (WD + Al): sensitive (Star) and resistant (Legacy). Plants were exposed to WD + Al conditions and treated with foliar applications of methyl jasmonate (MeJA) at concentrations of 10, 50, and 100 µM. Data correspond to the mean ± standard deviation (SD) of five biological replicates per treatment. Distinct lowercase letters indicate significant differences (Tukey’s test, *p* ≤ 0.05) between treatments within the same cultivar and sampling time; and capital letters indicate significant differences (Tukey’s test, *p* ≤ 0.05) between sampling times within the same treatment and cultivar.

The WD + Al stress condition significantly affected carotenoid accumulation in both cultivars of *V. corymbosum* (Figure 2). In the Star cultivar, WD + Al stress significantly increased total carotenoid content at 7 and 21 days of treatment compared with control plants. Notably higher contributions of violaxanthin and lutein were observed. Foliar MeJA application further enhanced carotenoid accumulation in Star, with the 50 µM dose producing the most pronounced effect at 7 days, increasing violaxanthin and β-carotene levels significantly above those observed under WD + Al alone. At 21 days, all MeJA doses maintained higher carotenoid levels than WD + Al alone, with increases primarily driven by lutein and violaxanthin. In the WD + Al-resistant cultivar, WD + Al stress also induced carotenoid accumulation, but the effect was less pronounced than in the WD + Al-sensitive cultivar. Foliar MeJA application notably increased carotenoid concentrations in the Star cultivar (WD + Al-sensitive), particularly at 7 days, with the 50 µM doses leading to significant increases in lutein and β-carotene levels. At 21 days, MeJA application sustained elevated carotenoid levels, mainly in the Legacy cultivar (WD + Al-resistant), with the 50 µM dose being the most effective.

### 2.2. Foliar MeJA Application Effect on Osmolyte Levels

**Figure 3:**
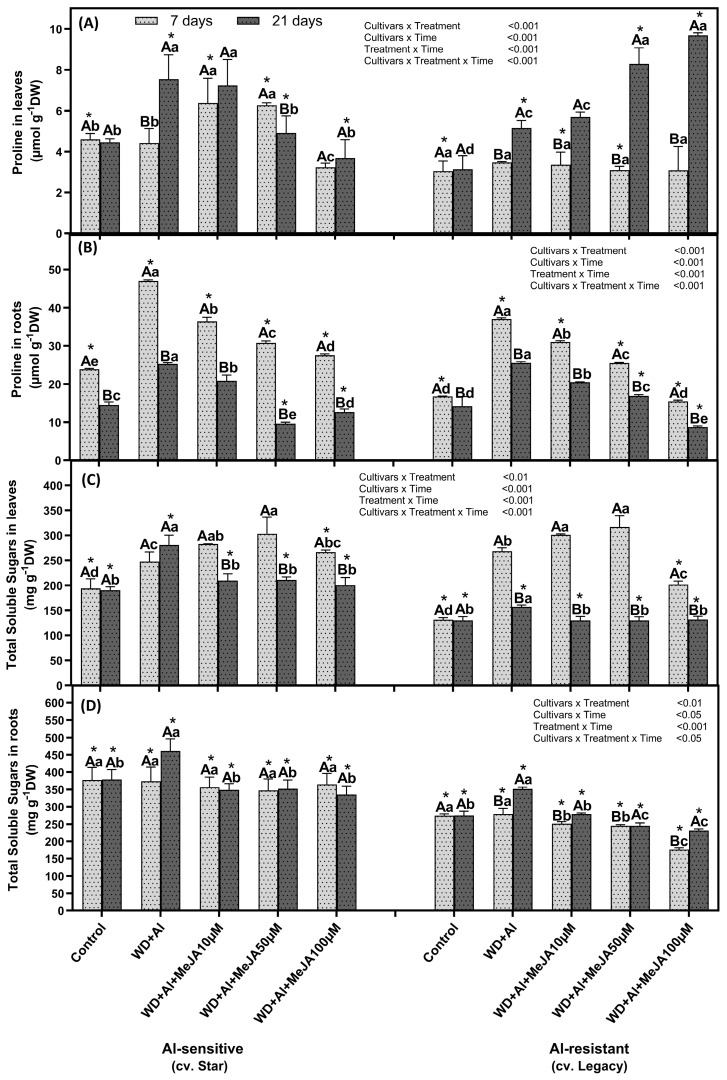
Proline and total soluble sugars in *V. corymbosum* cultivars exhibiting differential tolerance to combined water deficit and aluminum (WD + Al) stress: Sensitive (Star) and resistant (Legacy). Plants were subjected to WD + Al and treated with methyl jasmonate (MeJA) at concentrations of 10, 50, and 100 µM. Panels (**A**,**B**) show proline levels in leaves and roots, respectively, while panels (**C**,**D**) display total soluble sugar content in the same tissues. Data correspond to the mean ± standard deviation (SD) of five biological replicates per treatment. Distinct lowercase letters indicate significant differences (Tukey’s test, *p* ≤ 0.05) between treatments within the same cultivar and sampling time; capital letters indicate significant differences (Tukey’s test, *p* ≤ 0.05) between sampling times within the same treatment and cultivar; and asterisks (*) highlight significant differences (Tukey’s test, *p* ≤ 0.05) between cultivars under the same treatment and sampling point.

An increase in proline concentration was observed in both leaves and roots of both cultivars under WD + Al stress compared to the control treatment (Figure 3). In leaves, this increase was observed after 21 days of treatment in plants exposed to WD + Al stress, reaching up to 1.6 times higher than control plants in both cultivars. In the roots of the WD + Al-resistant cultivar, this increase was observed after 7 and 21 days under WD + Al stress, reaching 2 and 1.2 times higher, respectively, than in control plants (Figure 3). With respect to the MeJA application, at 21 days of treatment, a positive linear trend was observed in leaves of the WD + Al-resistant cultivar, with proline levels increasing proportionally with MeJA concentration (Figure S1).

With respect to the concentration of soluble sugars in leaves at 7 days, an increase was observed in plants under WD + Al treatments compared to the control, which was 2 times more in the WD + Al-resistant cultivar and 1.3 times more in the WD + Al-sensitive cultivar (Figure 3). Under the same conditions, both cultivars showed approximately 1.2-fold increases in their roots after 21 days of treatment. With respect to the MeJA application, an increase in total sugar concentrations in leaves of the resistant cultivar was observed at 7 days with the 10 and 50 μM doses, reaching up to 1.2 times higher than of plants treated with WD + Al without MeJA application (Figure 3). In this regard, no significant differences were observed in the susceptible cultivar following MeJA application in either time period (Figure 3).

### 2.3. Effect of MeJA Foliar Application on Organic Acid Levels

**Figure 4:**
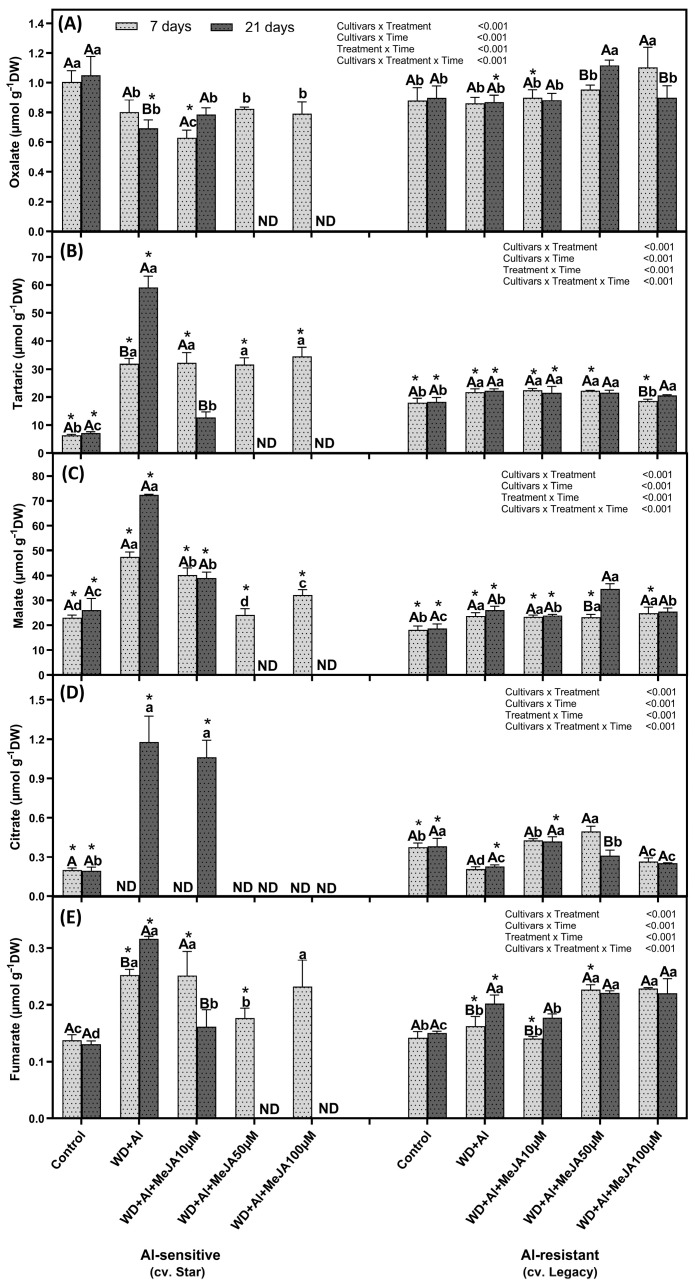
Oxalate (**A**), tartaric (**B**), malate (**C**) citrate (**D**) and fumarate (**E**) in leaves of *V. corymbosum* cultivars exhibiting differential tolerance to combined water deficit and aluminum (WD + Al) stress: Sensitive (Star) and resistant (Legacy). Plants were subjected to WD + Al and treated with methyl jasmonate (MeJA) at concentrations of 10, 50, and 100 µM. Data correspond to the mean ± standard deviation (SD) of five biological replicates per treatment. Distinct lowercase letters indicate significant differences (Tukey’s test, *p* ≤ 0.05) between treatments within the same cultivar and sampling time; capital letters indicate significant differences (Tukey’s test, *p* ≤ 0.05) between sampling times within the same treatment and cultivar; and asterisks (*) highlight significant differences (Tukey’s test, *p* ≤ 0.05) between cultivars under the same treatment and sampling point.

A significant increase in tartaric, malate, and fumarate concentrations was observed in leaves of both cultivars under WD + Al stress, compared to control plants, at 7 and 21 days of treatment (Figure 4). In addition, in leaves of the WD + Al-sensitive cultivar, a significant decrease in oxalate levels was observed at 7 and 21 days of treatments, whereas in the WD + Al-resistant cultivar, a reduction in citrate concentrations was observed at both time points in WD + Al compared to control plants. The MeJA application increased organic acid concentrations in the leaves of both cultivars above levels observed in control plants. In addition, in the WD + Al-resistant cultivar, MeJA mitigated the decrease in citrate induced by combined WD + Al stress at 10 and 50 µM, reaching values similar to those of the control plants. It is highlighted that in the leaves of the Star cultivar, none of the quantified organic acids were detected at 21 days, mainly at the higher MeJA dose; whereas in the resistant cultivar (Legacy), organic acids were detected at both time points (Figure 4).

**Figure 5:**
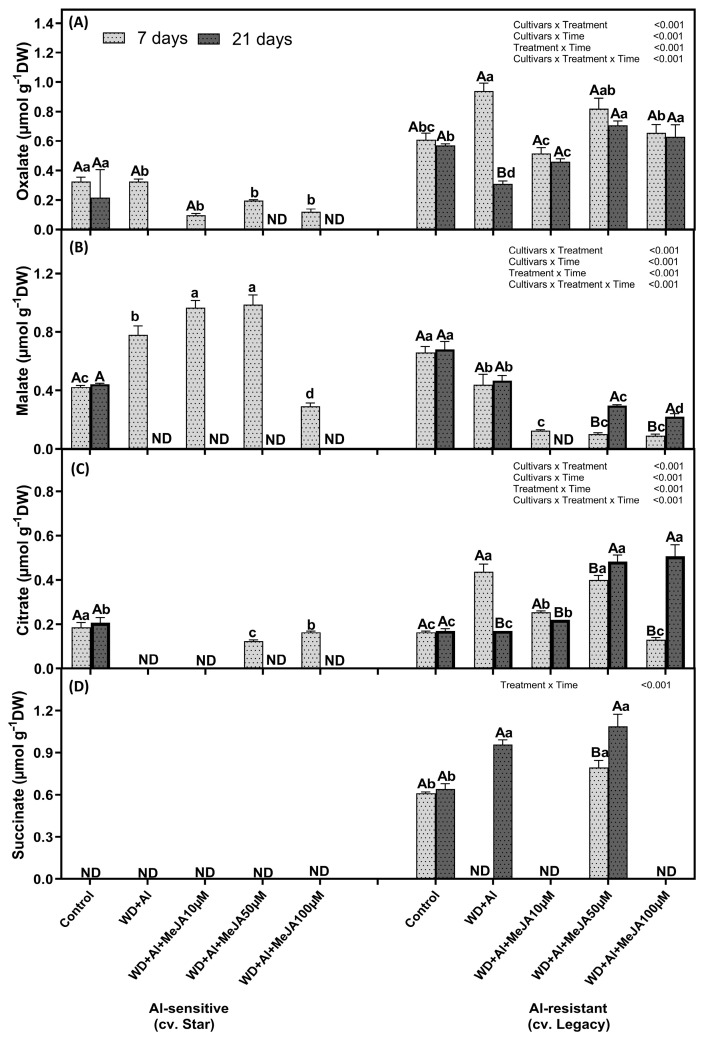
Oxalate (**A**), malate (**B**), citrate (**C**), and succinate (**D**) in roots of *V. corymbosum* cultivars exhibiting differential tolerance to combined water deficit and aluminum (WD + Al) stress: Sensitive (Star) and resistant (Legacy). Plants were subjected to WD + Al and treated with methyl jasmonate (MeJA) at concentrations of 10, 50, and 100 µM. Data correspond to the mean ± standard deviation (SD) of five biological replicates per treatment. Distinct lowercase letters indicate significant differences (Tukey’s test, *p* ≤ 0.05) between treatments within the same cultivar and sampling time; and capital letters indicate significant differences (Tukey’s test, *p* ≤ 0.05) between sampling times within the same treatment and cultivar.

The combined WD + Al stress significantly affected the concentrations of organic acids in the roots of both cultivars (Figure 5). In the WD + Al-sensitive cultivar, increases in oxalate and malate were observed at 7 days, but these compounds were undetectable at 21 days. In contrast, the WD + Al-resistant cultivar (Legacy) showed significant increases in oxalate, malate, and citrate at 7 and 21 days. Foliar MeJA application enhanced organic acid concentrations in Legacy, especially at the 50 µM dose at 21 days, exceeding levels observed under WD + Al without MeJA. Succinate was detected only in the control, WD + Al, and WD + Al + MeJA10µM of the Legacy cultivar, mainly at 21 days (Figure 5).

### 2.4. Impact of Foliar MeJA Application on Auxin Levels in Leaves and Roots

**Figure 6:**
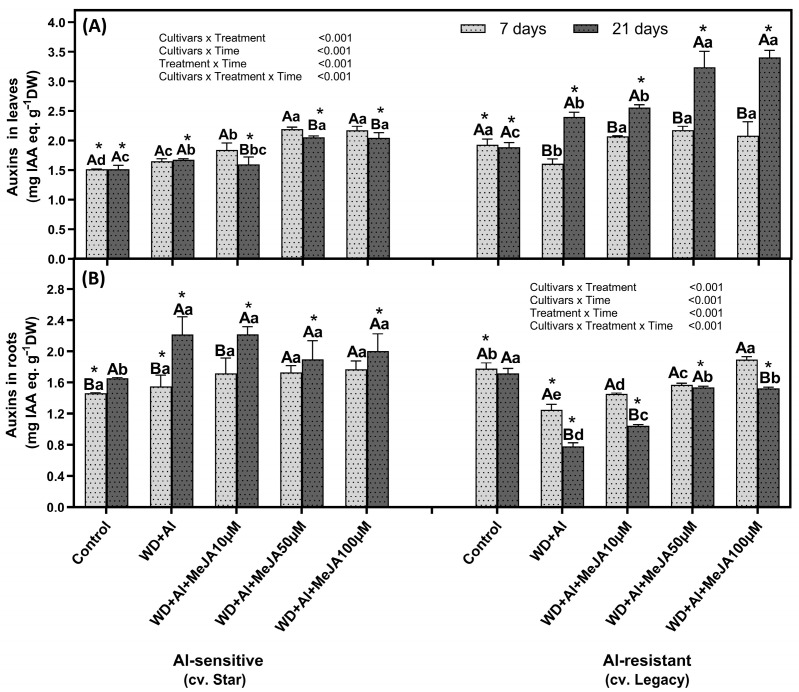
Auxins concentration in leaves (**A**) and roots (**B**) of *V. corymbosum* cultivars exhibiting differential tolerance to combined water deficit and aluminum (WD + Al) stress: sensitive (Star) and resistant (Legacy). Plants were subjected to WD + Al and treated with methyl jasmonate (MeJA) at concentrations of 10, 50, and 100 µM. Data correspond to the mean ± standard deviation (SD) of five biological replicates per treatment. differences are indicated as follows: distinct lowercase letters indicate significant differences (Tukey’s test, *p* ≤ 0.05) between treatments within the same cultivar and sampling time; capital letters indicate significant differences (Tukey’s test, *p* ≤ 0.05) between sampling times within the same treatment and cultivar; and asterisks (*) highlight significant differences (Tukey’s test, *p* ≤ 0.05) between cultivars under the same treatment and sampling point.

In the Star cultivar, a significant increase in AUX concentration was observed in leaves and roots of plants under combined WD + Al stress compared to control plants, at both treatment times (Figure 6). This increase was more pronounced in roots at 21 days, reaching up to 2.3 times the levels observed in control plants. In the Legacy cultivar, under WD + Al stress, leaves showed a decrease in AUX at 7 days (1.2 times lower than control plants) and a significant increase at 21 days (1.2 times higher). In roots, AUX concentration decreased at 7 days (1.4-fold lower) and was further reduced at 21 days (2.2-fold lower than control plants). Foliar application of MeJA increased AUX concentration in WD + Al stressed plants. In Star, at 7 days, all MeJA doses significantly increased AUX concentration in leaves, reaching up to 1.3 times higher than in plants under WD + Al without MeJA. At 21 days, this increase was evident only with the 50 and 100 µM doses, reaching up to 1.2-fold. In Legacy leaves at 21 days, the MeJA application increased AUX concentrations by 1.4-fold compared to plants under WD + Al without MeJA. This effect was observed in roots at both time points, with up to a 2-fold increase (Figure 6).

### 2.5. Foliar MeJA Application Effect on the Gene Expressions Associated with Aluminium Resistance Mechanisms

**Figure 7:**
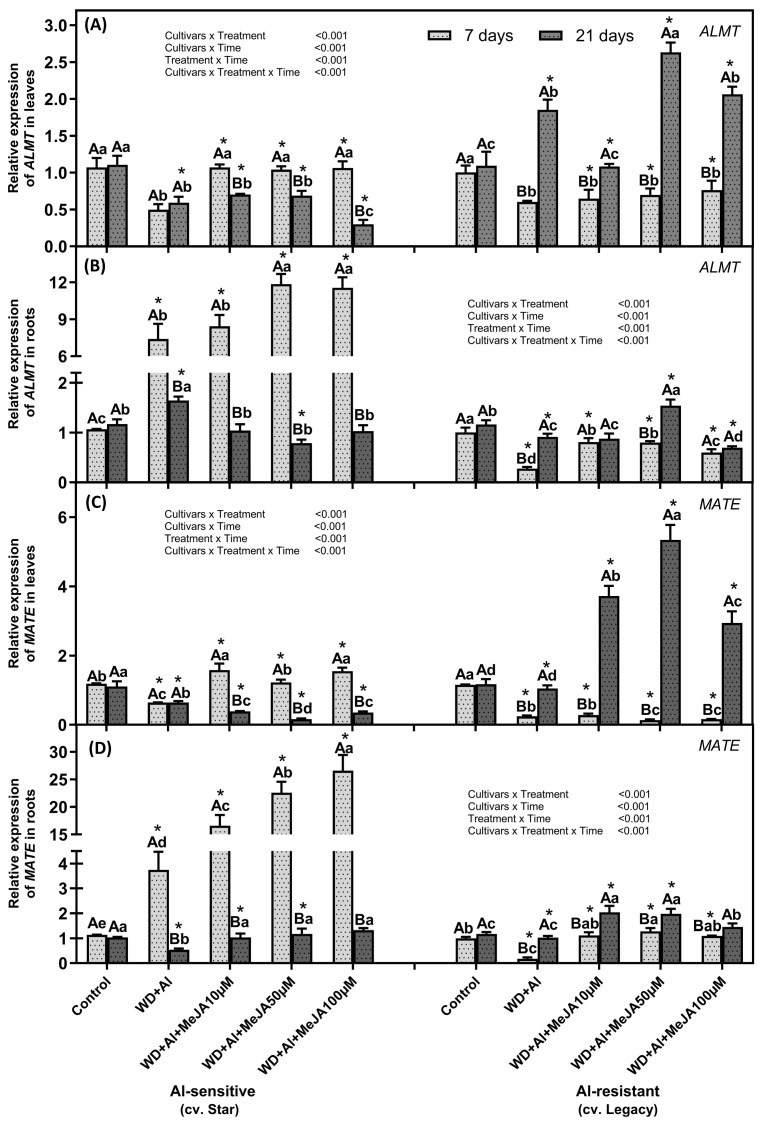
Relative expression of *ALMT* and *MATE* genes in leaves (**A**,**C**) and roots (**B**,**D**) of *V. corymbosum* cultivars with contrasting sensitivity to combined water deficit and aluminum (WD + Al) stress: sensitive (Star) and resistant (Legacy). Plants were subjected to WD + Al and treated with methyl jasmonate (MeJA) at concentrations of 10, 50, and 100 µM. Panels A and B show *ALMT* transcript levels in leaves and roots, respectively; while panels C and D show *MATE* gene expression in the same tissues. Data correspond to the mean ± standard deviation (SD) of five biological replicates per treatment. Distinct lowercase letters indicate significant differences (Tukey’s test, *p* ≤ 0.05) between treatments within the same cultivar and sampling time; capital letters indicate significant differences (Tukey’s test, *p* ≤ 0.05) between sampling times within the same treatment and cultivar; and asterisks (*) highlight significant differences (Tukey’s test, *p* ≤ 0.05) between cultivars under the same treatment and sampling point.

In the WD + Al-sensitive cultivar, after 7 days of treatment under WD + Al stress, *ALMT* and *MATE* genes were up-regulated in roots, whereas leaves were down-regulated (Figure 7). In contrast, in the WD + Al-resistant cultivar, *ALMT* genes were up-regulated in leaves and down-regulated in roots compared with control plants at 21 days of treatment. Foliar application of MeJA significantly increased in *ALMT* and *MATE* expression in plants under WD + Al stress. In the WD + Al-sensitive cultivar, at 7 days, MeJA induced an up-regulation of *ALMT* and *MATE* in both leaves (up 2- and 2.4-fold, respectively) and roots (up 1.6- and 7.1-fold, respectively) relative to plants stressed without MeJA application. At 21 days, only *MATE* showed up-regulation in roots, with increases of up to 2.4-fold across all MeJA doses. In the WD + Al-resistant cultivar, at 7 days, no significant changes in *ALMT* and *MATE* expression were observed in leaves, but in roots, *ALMT* increased up to 2.9-fold (10 and 50 µM doses) and *MATE* up to 7.2-fold with all doses. At 21 days, MeJA application significantly increased the expression of both genes in leaves (*ALMT*: 1.4-fold; *MATE*: 5.1-fold with 50 µM) and roots (*ALMT*: 1.7-fold with 50 µM; *MATE*: up to 1.9-fold with 10 and 50 µM) compared to stressed plants without MeJA (Figure 7).

### 2.6. Multivariate Analysis

**Figure 8:**
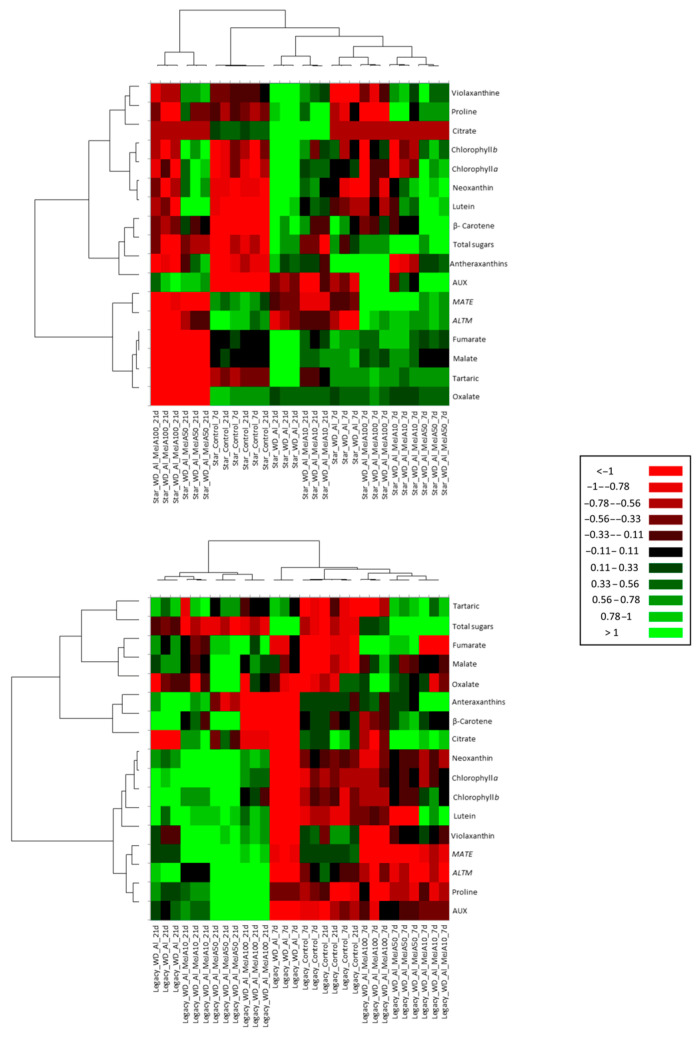
Heatmap-based hierarchical clustering of leaf samples from *V. corymbosum* cultivars with differing tolerance to combined water deficit and aluminum stress (WD + Al): Star (sensitive) and Legacy (resistant). Plants were treated with methyl jasmonate (MeJA) at 10, 50, and 100 μM under WD + Al conditions. The green color indicates higher significance, and red color indicates lower significance. Hierarchical clustering was performed using Euclidean distances, with the mean as the metric parameter and the linking method.

**Figure 9:**
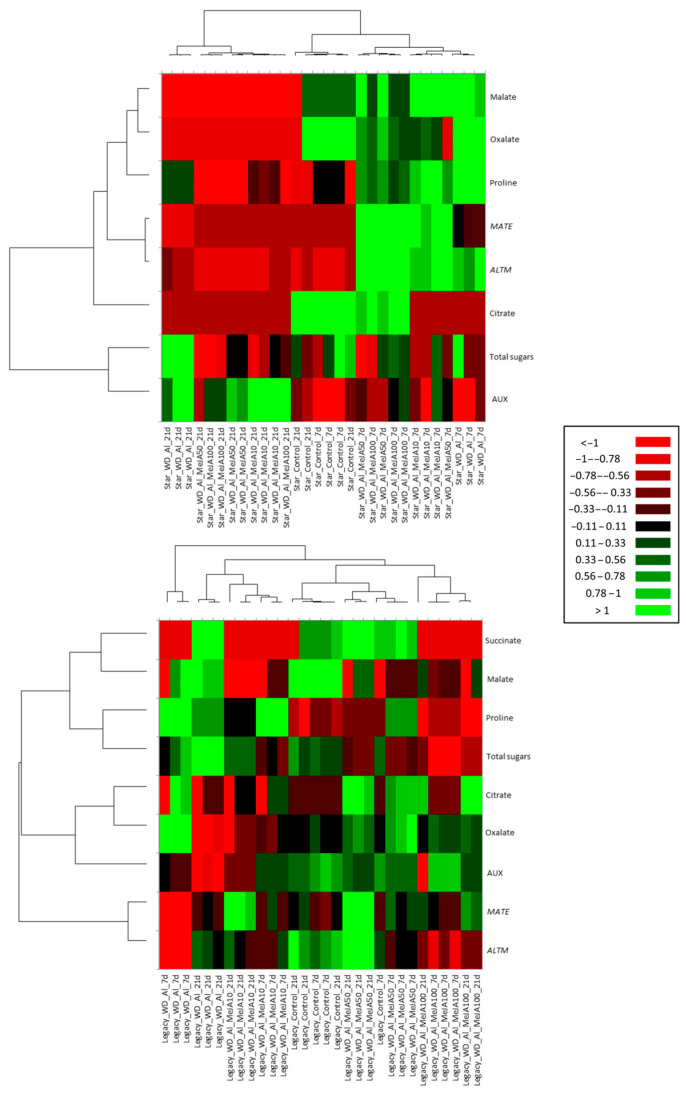
Heatmap-based hierarchical clustering of roots samples from *V. corymbosum* cultivars with differing tolerance to combined water deficit and aluminum stress (WD + Al): Star (sensitive) and Legacy (resistant). Plants were treated with methyl jasmonate (MeJA) at 10, 50, and 100 μM under WD + Al conditions. The green color indicates higher significance, and red color indicates lower significance. Hierarchical clustering was performed using Euclidean distances, with the mean as the metric parameter and the linking method.

Hierarchical clustering revealed distinct responses to combined WD + Al stress among *V. corymbosum* cultivars, reflecting the temporal and organ-specificity of resistance mechanisms (Figure 8). In leaves, Star showed higher significance (green) in the accumulation of photosynthetic pigments (Antheraxanthins) and organic acids (malate, fumarate, and tartaric). On the contrary, Legacy showed lower significance (red) in these initial responses but stood out in the late stages, with a sustained increase in the accumulation of proline, AUX, organic acids, and pigments, along with increased in *ALMT* and *MATE* gene expression. In roots, Star showed greater significance in malate, oxalate, proline, and *ALMT* expression at 7 days, while Legacy presented, at 21 days, remarkable accumulations of oxalate, malate, succinate, carbohydrates, and proline (Figure 9).

## 3. Discussion

### 3.1. Exogenous MeJA Enhances Chlorophyll and Carotenoid Accumulation, Improving Resistance to Combined WD + Al Stress in V. corymbosum

Photosynthetic performance is highly dependent on the content and function of photosynthetic pigments, as they play a key role in capturing solar energy, facilitating its conversion into chemical energy, and storing it in plant biomass [29]. Our results indicate that in *V. corymbosum* plants exposed to the combined WD + Al stress, chlorophyll and carotenoid content were reduced at 7 days of treatment in the cultivar Legacy (Figure 1 and Figure 2). This decrease was mitigated by MeJA foliar application, reaching values even higher than those observed in control plants. This behavior contrasts with that reported in *V. corymbosum* plants subjected only to WD stress, where no significant differences in photosynthetic pigment accumulation were detected between plants stressed with and without MeJA application at 7 days of treatment [6]. However, in plants subjected only to Al stress, a pattern similar to that in our study was observed, in which foliar application of MeJA increased photosynthetic pigment accumulation at 48 h of treatment compared to plants exposed to Al without MeJA treatment [30]. According to studies conducted on wheat plants subjected to WD stress and treated with MeJA, it is established that MeJA induces chlorophyll accumulation, probably due to the decrease in the degradation of these pigments by chlorophyllase and peroxidase enzymes [31]. Also, Esmaielzadeh et al. [32] have reported that increases in photosynthetic pigments associated with foliar application of MeJA under Al stress correlate with increases in non-protein thiol compounds (NPT), which, by sequestering Al in roots and reducing its translocation to the shoot, protect photosynthetic organs against toxic metal damage and promote plant growth under Al toxicity conditions. Taken together, our results demonstrate that foliar application of MeJA effectively mitigates the negative effects of combined WD and Al stresses in *V. corymbosum*, maintaining and even increasing photosynthetic pigment accumulation.

After 21 days of treatment, an increase in photosynthetic pigments, both chlorophylls and carotenoids, was observed, an effect enhanced by foliar MeJA application, mainly at the 50 μM dose. Chlorophyll (Chl) content, especially Chl b, influences the size of the light-harvesting antenna, possibly due to increased formation of light-harvesting Chl a/b protein complexes (LHCs), which would increase the size of peripheral antennae and optimize photosynthetic parameters [14]. Our results, together with those of Cáceres et al. [7], support this observation since the increase in chlorophylls was directly associated with the stability of photosynthetic parameters, particularly in the WD + Al-resistant cultivar. With respect to carotenoids, a significant increase in neoxanthins and anteraxanthins was observed in the WD + Al-resistant cultivar. These carotenoids are of great importance as they participate in the dissipation of excess light energy by a process called non-photochemical thermal dissipation (NPQ) [15]. In this process, it is known that under high light conditions, the protonated Photosystem II Subunit S (PsbS) protein and the conversion of violaxanthin to zeaxanthin form complexes that convert light-harvesting systems into heat sinks [33]. These results underscore the role of MeJA as a key modulator in protecting the photosynthetic apparatus and enhancing abiotic stress resistance in *V. corymbosum*.

### 3.2. Foliar Application of MeJA Modulates the Osmolytes Levels, Favoring Resistance to WD + Al Stress in V. corymbosum

A resistance mechanism used by plants to maintain their water status is the accumulation of osmolytes, which help maintain turgor pressure and relative water content (RWC), facilitating their adaptation to conditions of limited water availability [19]. Recently, Cáceres et al. [7] demonstrated that *V. corymbosum* plants under combined WD + Al stress and treated with MeJA maintain a relative water content (RWC) similar to the control. This result is associated with the significant increase in proline and total soluble sugars observed in both leaves and roots of both cultivars under combined WD + Al stress compared to the control. Moreover, these results agree with those reported by Slugeňová et al. [34], who observed that in *Picea abies* subjected to combined WD + Al stress, proline concentration was 4.95 times higher than in control plants.

With respect to foliar application of MeJA, in the WD + Al-resistant cultivar, it significantly increased osmolyte accumulation compared to plants subjected to combined WD + Al stress without MeJA, with an early (7 days) increase in carbohydrates and a late (21 days) increase in proline. This finding aligns with that reported by Fugate et al. [35], who identified that in *Beta vulgaris* L. exposed to WD stress and treated with MeJA, proline accumulation was 2.6- to 3.3-fold higher than in untreated plants. However, it is not known what mechanism MeJA uses to induce increased osmolyte accumulation. According to Zivcak et al. [36], osmolyte accumulation may be regulated by increased biosynthesis, decreased degradation, or increased or decreased osmolyte transport. Thus, proline accumulation is regulated by the enzymes pyrroline-5-carboxylate reductase (P5CR) and proline dehydrogenase (ProDH), which are responsible for its biosynthesis and degradation, respectively, as well as by amino acid transporters (ATFs) [37,38]. On the other hand, the accumulation of soluble sugars is regulated through triose production from the Calvin-Benson cycle, starch degradation, and transport mediated by sugar transporters (SWEETs), sucrose transporters (SUTs), and monosaccharide transporters (MSTs) [39,40]. Taken together, these results suggest that exogenous application of MeJA contributes to foliar osmotic adjustment under combined WD + Al stress by temporally modulating key osmolytes such as proline and sugars.

### 3.3. MeJA Enhances Differential Accumulation of Organic Acids by Regulating ALMT and MATE Transporters in Response to Combined WD + Al Stress in V. corymbosum

Organic acids contribute to Al detoxification in plants by excluding Al from the rhizosphere, forming non-toxic complexes, intracellularly chelating Al to reduce cytoplasmic toxicity, and sequestering Al in the vacuole, preventing metabolic damage [22]. Under combined WD + Al stress, *V. corymbosum* cultivars show differential accumulation of organic acids and variable expression of genes encoding *ALMT* and *MATE* transporter proteins in both leaves and roots. Under combined WD + Al stress, organic acids accumulated predominantly in leaves, with significantly higher accumulation of tartaric, malate, citrate, and fumarate in the Star cultivar compared to Legacy. In contrast, in roots under the same stress conditions, Legacy showed greater accumulation of organic acids, with significant increases in oxalate, citrate, and succinate; whereas in Star, only malate showed significant increases. Similar results were reported by Cárcamo-Fincheira et al. [5], who observed that the cultivar Star, under individual Al stress, presented a high concentration of malate in leaves and roots, significantly higher than that of some WD + Al-resistant cultivars.

Furthermore, our results reveal that the increase in malate in Star leaves was associated with a significant decrease in *ALMT* expression, which explains the intracellular accumulation in leaves. Accumulation of organic acids, both under single Al stress and combined WD + Al, has been identified as a key mechanism of resistance, as these compounds bind to Al, forming non-toxic complexes that accumulate in vacuoles of leaves, roots, and other organs such as trichomes [41,42]. Furthermore, after 7 days of WD + Al treatment, increases in *ALMT* and *MATE* expression were observed in the WD + Al-sensitive cultivar, which were significantly higher with foliar application of MeJA. These results align with previous research showing that jasmonates (JAs) increase the exudation of organic acids, such as malate. Wang et al. [21] determined that the presence of Al in the growth medium increases endogenous JAs levels in tomatoes, thereby intensifying *ALMT* gene expression, facilitating malate transport, and promoting Al detoxification.

### 3.4. MeJA-Induced Auxin Accumulation May Be Involved in the Regulation of Organic Acid Transport

The mechanism by which foliar MeJA application stimulates these resistance mechanisms could involve regulating endogenous phytohormonal signaling. The application of MeJA at different stages of plant development or under abiotic stress conditions has been shown to stimulate AUX biosynthesis [24,25,26]. This agrees with the results of this study, which show that foliar MeJA application induces AUX accumulation in plants subjected to WD + Al stress (Figure 7). Also, a correlation was observed between AUX accumulation and increased expression of *ALMT* and *MATE* genes, manifesting at 7 days in roots of the WD + Al-sensitive cultivar and at 21 days in leaves of the WD + Al-resistant cultivar, an effect enhanced by the MeJA application. Therefore, it is suggested that MeJA induces AUX accumulation to promote the transport of organic acids, such as malate and citrate, at different times depending on the stress resistance of the cultivar. This hypothesis is consistent with the findings of other researchers. Thus, in studies on Al toxicity in plants, it has been reported that an increase in endogenous AUXs in the roots of *Triticum aestivum* L. could activate Mitogen-Activated Protein Kinase-type (MAPK) proteins, which in turn activate anion channels, through which malate transport could be facilitated [27,28].

### 3.5. The WD + Al-Sensitive Cultivar Responds Earlier to the Combined WD + Al Stress than the Resistant Cultivar

According to the three-way statistical analyses, a significant interaction between the factors was observed, indicating that the two *V. corymbosum* cultivars respond differently to the time and treatments. This result agrees with the hierarchical cluster analysis, which showed differential activation of resistance mechanisms in *V. corymbosum* cultivars under combined WD + Al stress (Figure 8 and Figure 9). The WD + Al-sensitive cultivar (Star) exhibited an early response at 7 days, evidenced by a significant increase in the accumulation of total sugars, carotenoids, and organic acids in both leaves and roots, with the increase in leaves persisting until 21 days of treatment. At the same time, the resistant cultivar (Legacy) showed a more sustained and delayed response at 21 days, which was further intensified by the application of 50 μM MeJA. These results are consistent with previous studies on *V. corymbosum* under individual Al stress, in which the Star cultivar exhibited a higher antioxidant and organic acid accumulation, including malate, at 7 days of treatment. In contrast, the resistant cultivars (Camellia and Cargo) showed this response later, at 21 days [5]. Overall, these results suggest that Al sensitivity is associated with rapidly activated resistance mechanisms, whereas Al resistance is associated with more sustained strategies over time.

## 4. Materials and Methods

### 4.1. Soil, Plant Material, and Growing Conditions

The experimental setup was established using an Andisol-type soil collected in the Lastarria area of the Araucanía Region, Chile. According to CIREN [43], this soil naturally has low aluminum levels (65 mg kg^−1^). Two cultivars of *V. corymbosum* with known differential responses to combined WD + Al stress were selected: Star (sensitive to WD + Al stress) and Legacy (resistant to WD + Al stress), as reported by Cáceres et al. [7]. These cultivars were purchased from Global Seedling SpA in the Maule Region. Seedlings of uniform size (30 to 40 cm in height) were selected and transplanted into plastic pots containing 1 kg of Andisol. The plants were acclimatized under controlled greenhouse conditions for two weeks, as described by Cáceres et al. [7]. Soil moisture levels were monitored with calibrated sensors to ensure irrigation at 80% of field capacity (FC). The FC value was previously determined using the method described by Brischke and Wegener [44], which employs a cylindrical sand bath to evaluate soil water retention.

### 4.2. Applied Treatments and Experimental Design

Following acclimation, control plants continued receiving irrigation to maintain 80% FC. For water deficit (WD) conditions, irrigation was withheld until the substrate reached 50% FC, as described by Almutairi et al. [45] and Chen et al. [46]. To impose aluminum toxicity, plants under WD conditions were treated with an aluminum chloride (AlCl_3_) solution (18% *w*/*v*). A volume of 10 mL AlCl_3_ per kilogram of soil was applied via irrigation to achieve an Al concentration of 1665 mg kg^−1^, as described by Slugeňová et al. [34]. Subsequently, exogenous methyl jasmonate (MeJA) was applied foliarly at three different concentrations: 10, 50, and 100 µM. Each MeJA solution was prepared using 0.05% Tween 80 as a surfactant and applied in the early morning hours to maximize absorption efficiency. Leaf and root tissues were sampled at 7 and 21 days after MeJA application. Therefore, the experimental design included three main factors: cultivar (Star and Legacy), time (7 and 21 days), and treatments (Control, WD + Al, WD + Al + MeJA10µM, WD + Al + MeJA50µM, and WD + Al + MeJA100µM), as shown in Supplementary Table S1.

### 4.3. Determination of Pigment Levels

The extraction of chlorophylls and carotenoids from lyophilized leaves was conducted using pure acetone (100% *v*/*v*, HPLC grade) according to the method outlined by García-Plazaola and Becerril [47]. The analysis was performed via high-performance liquid chromatography (HPLC) on an Agilent Technologies 1200 series system, employing a Waters Spherisorb C-18 column (5.0 µm ODS1, 4.6 × 250 mm). Standard pigments from Sigma-Aldrich (Sigma Chemical Co., St. Louis, MO, USA) like violaxanthin (Vx), antheraxanthin (Ax), zeaxanthin (Zx), neoxanthin (Nx), chlorophyll a (Chl a), chlorophyll b (Chl b), β-carotene (βCa), and lutein (Lt) were used.

### 4.4. Osmolytes Determination

Proline was determined following the methodology proposed by Carillo and Gibon [48]. Leaf and root samples were harvested at the beginning of the light period (between 8:00 and 10:00 h), snap frozen in liquid nitrogen, and stored at −80 °C. The frozen leaves were then lyophilized for proline extraction. To carry out the proline extraction, the tissue was first macerated with liquid nitrogen, and then three consecutive extractions were performed with ethanol at 100, 80, and 50% *v*/*v*, respectively. An aliquot of 0.1 mL of extract was then added to 0.1 mL of a 1% (*w*/*v*) ninhydrin solution in 60% (*v*/*v*) acetic acid. After homogenization, the mixture was heated at 95 °C for 20 min. After cooling, the absorbance was determined at 520 nm. Proline concentration was expressed as mg g^−1^ dry weight (DW).

Total soluble sugars were quantified following the method proposed by Roe [49], using 0.1 g of lyophilized leaves. The measure was performed at 520 nm using a microplate reader, and the concentration of total sugars was expressed as mg g^−1^ dry weight (DW) [50].

### 4.5. Organic Acid Determinations

Extraction and quantification of organic acids were carried out using leaves and roots of *V. corymbosum* frozen in liquid nitrogen, freeze-dried, and ground to a fine powder. Approximately 15 mg of the powdered tissue was placed in 1.5 mL tubes and mixed with 1 mL of 80% (*v*/*v*) methanol. Samples were incubated in a water bath at 70 °C for 10 min with occasional shaking, then centrifugated at 10,000 rpm for 10 min at 4 °C. The supernatant was carefully removed, and the extraction process was repeated twice to improve the recovery of organic acids [51]. The combined supernatants were filtered through a 0.22 µm membrane before injection into the HPLC system. Quantification was performed on an HPLC system equipped with a UV-Vis detector and a C18 reversed-phase column, using a mobile phase of 0.1% phosphoric acid in water with a flow rate of 1 mL min^−1^ and detection at 210 nm. Twenty microliters of each sample were injected, and the peaks corresponding to oxalate, tartaric, malate, citrate, succinate, and fumarate were identified by comparing their retention times with those of commercial standards. The absolute concentrations were determined using standard calibration curves [52].

### 4.6. Leaf and Roots Auxins Concentration

A solvent-induced phase transition extraction (SIPTE) was used on freeze-dried leaf and root samples to quantify AUXs, as described by Liu et al. [53]. Then, samples were concentrated using an integrated vacuum concentrator system (Savant SPD11V) and resuspended in 1 mL of 30% *v*/*v* MeOH. The total AUX concentration was estimated using the Salkowski method as outlined by Sarwar and Kremer [54], with some adaptations for highbush blueberries. A 200 μL of the sample and 50 μL of the Salkowski reagent were used, followed by a 25 min incubation at room temperature. Subsequently, absorbance was read at 530 nm using a plate reader. The Salkowski reagent was prepared according to the instructions of Glickmann and Dessaux [55]. Indole acetic acid (IAA) was used as the standard, and the total AUX concentration was expressed in mg IAA eq g^−1^ DW.

### 4.7. Transcript Analyses by Quantitative Real-Time PCR (qRT-PCR)

The RNA from leaves and roots was extracted using the method described by Jaakola et al. [56]. This method involves an extraction buffer containing CTAB (2% *w*/*v*) to separate polysaccharides from nucleic acids, as well as PVP (2% *w*/*v*) and β-mercaptoethanol (2% *v*/*v*) to reduce oxidation of phenolic compound. To purify the extract, a series of Liquid–Liquid extractions was performed with chloroform–isoamyl alcohol (24:1), and 10 M LiCl and Isopropanol were used as precipitating agents to obtain RNA in pellet form. The pellet was then resuspended in H_2_O DEPC. RNA concentrations and purity were measured using a Varioskan Flash μDropTM Plate multimode spectral reader (Thermo Scientific, Wilmington, DE, USA), while RNA quality was visually assessed by denaturing RNA gel electrophoresis.

Following the manufacturer’s recommendations, first-strand cDNA was synthesized from total RNA isolated from leaves and roots. The relative quantification of the *Aluminum-Activated Citrate Transporter* (*MATE*) and *Aluminum-Activated Malate Transporter* (*ALMT*) genes was conducted using the *metallothionein* (*MET*) reference gene, as described by Naik et al. [57] and Zifkin et al. [58]. Primers for these genes were designed based on assembled *Vaccinium* transcripts (Table 1). Quantification of gene expression was performed using real-time PCR (Applied BiosystemsTM QuantStudioTM 3 Real-Time PCR System). Gene expression data (Ct values) were utilized for relative gene expression quantification using the comparative 2^−ΔΔCt^ method [59].

### 4.8. Statistical Analysis

The experimental setup was structured as a factorial design incorporating three fixed factors: Cultivars (Star and Legacy), time (7 and 21 days), and treatment condition (five levels) (see Supplementary Table S1). Each treatment combination was evaluated using five independent biological replicates and three technical replicates for each physiological, biochemical, and molecular measurement. Prior to conducting parametric tests, datasets were examined to confirm adherence to the assumptions of normality (using the Shapiro–Wilk test) and homogeneity of variances (using Levene’s test). A three-way analysis of variance (ANOVA) was employed to determine the significance of main effects and interactions among the experimental factors. When significant differences were detected, a Tukey test was performed with a significance threshold set at *p* ≤ 0.05. All statistical analyses were performed using SigmaPlot version 12.0. Graphical representations were constructed using GraphPad Prism version 10.1.2. Additionally, to assess patterns of similarity across treatment responses, a hierarchical clustering analysis was conducted. For this purpose, the datasets were previously log-transformed (natural logarithms) to normalize variable scales. The heatmaps and clustering analyses were generated using XLSTAT-LifeScience 2024.

## 5. Conclusions

The results of this study confirm the hypothesis that foliar application of MeJA induced resistance mechanisms to the combined WD + Al stress in *V. corymbosum*. This mitigation was manifested through adjustments in the levels of photosynthetic pigments, osmolytes, organic acids, and phytohormones, in a cultivar- and time-dependent manner. First, it was evidenced that MeJA favors the accumulation of chlorophylls and carotenoids, contributing to maintaining photosynthetic efficiency, especially during the late stress phase in the resistant cultivar Legacy. In the case of the sensitive cultivar Star, the response was earlier but less sustained. Likewise, the increase in proline and soluble sugars under MeJA treatment supports its role as a key osmotic regulator, allowing the maintenance of water status under conditions of low water availability. Another relevant finding was the effect of MeJA on the accumulation and regulation of organic acids, such as malate, citrate, and oxalate, which participate in Al detoxification at both the foliar and root levels. This accumulation was accompanied by overexpression of *ALMT* and *MATE* transporter genes, especially in the Legacy cultivar, indicating molecular control coordinated by MeJA that enables the mobilization of organic acids. Finally, it was observed that MeJA-induced AUX accumulation correlated with the expression of organic acid transporters, suggesting a possible role of AUXs as positive regulators of these resistance mechanisms. Taken together, the results show that foliar application of MeJA activates defense mechanisms differentially according to cultivar sensitivity to WD + Al stress. While Star responds rapidly through metabolite accumulation and initial gene expression, Legacy shows a more sustained response, probably more efficient in the long term. These temporal and spatial differences in the activation of defense mechanisms suggest that MeJA may be an effective biotechnological tool for strengthening the resilience of *V. corymbosum* in acidic soil environments with limited water availability.

## Figures and Tables

**Table 1:** Primer sequence used in qRT-PCR, *ALMT* and *MATE* genes detected in leaves and roots of *V. corymbosum*.

Target Gene	Primer Name	Forward/Reverse
*MET*	Metallothionein	5′-ACC CTG ACA TGA GCT TCT CG-3′
		5′-ACC CAA ATC TCT GCT TGC TG-3′
*ALMT*	Aluminum-activated malate transporter	5′-GAG TTT GTG GCA AGG CTT CA-3′
		5′-ATT GCC CCT CTT TCT TCC CA-3′
*MATE*	Aluminum-activated citrate transporter	5′-TCA TGC TGG GAA TGG CTA GT-3′
		5′-GCT ATG TCT TCT GCT TGC CC-3′

## Data Availability

All data supporting the findings of this study are available within the paper.

## Supplementary Materials

The following supporting information can be downloaded at: https://www.mdpi.com/article/10.3390/plants14203202/s1, Figure S1: Correlation between Proline in leaves and treatments at 21 days; Table S1: Experimental design.

## Abbreviations

The following abbreviations are used in this manuscript:

WD	Water Deficit
MeJA	Methyl Jasmonate
*ALMT*	Aluminium-Activated Malate Transporter
*MATE*	Multidrug and Toxic Compound Extrusion
Ψw	Water Potential
RWC	Relative Water Content
RGR	Relative Growth Rate
Pn	Net Photosynthesis
gs	Stomatal Conductance
E	Transpiration Rate
LHC	Light-Harvesting Complexes
NPQ	Non-Photochemical quenching
OAs	Organic Acids
AUXs	Auxins
