# Supplementary material for: Differential Induction of Resistance Mechanisms by Methyl Jasmonate in Two Vaccinium corymbosum L. Cultivars Under Combined Water Deficit and Aluminum Toxicity
Source: Plants (Basel). 2025 Oct 18;14(20):3202. doi: 10.3390/plants14203202 (PMC12567442; doi:10.3390/plants14203202)
Supplement: Supplementary file 1 [file plants-14-03202-s001.zip › Table S1.pdf]

Cultivar	Evaluation time	Treatment	Irrigation conditions	Al saturation (%)	MeJA (µM)	Replication
Al- resistance	7 days	Control	80% FC	-	-	5
		WD+Al	50% FC	85	-	5
		WD+Al+MeJA 10	50% FC	85	10	5
		WD+Al+MeJA 50	50% FC	85	50	5
		WD+Al+MeJA 100	50% FC	85	100	5
	21 days	Control	80% FC	-	-	5
		WD+Al	50% FC	85	-	5
		WD+Al+MeJA 10	50% FC	85	10	5
		WD+Al+MeJA 50	50% FC	85	50	5
		WD+Al+MeJA 100	50% FC	85	100	5
Al-sensitive	7 days	Control	80% FC	-	-	5
		WD+Al	50% FC	85	-	5
		WD+Al+MeJA 10	50% FC	85	10	5
		WD+Al+MeJA 50	50% FC	85	50	5
		WD+Al+MeJA 100	50% FC	85	100	5
	21days	Control	80% FC	-	-	5
		WD+Al	50% FC	85	-	5
		WD+Al+MeJA 10	50% FC	85	10	5
		WD+Al+MeJA 50	50% FC	85	50	5
		WD+Al+MeJA 100	50% FC	85	100	5
