# Supplementary figures and images for: Differential Induction of Resistance Mechanisms by Methyl Jasmonate in Two Vaccinium corymbosum L. Cultivars Under Combined Water Deficit and Aluminum Toxicity
Source: Plants (Basel). 2025 Oct 18;14(20):3202. doi: 10.3390/plants14203202 (PMC12567442; doi:10.3390/plants14203202)

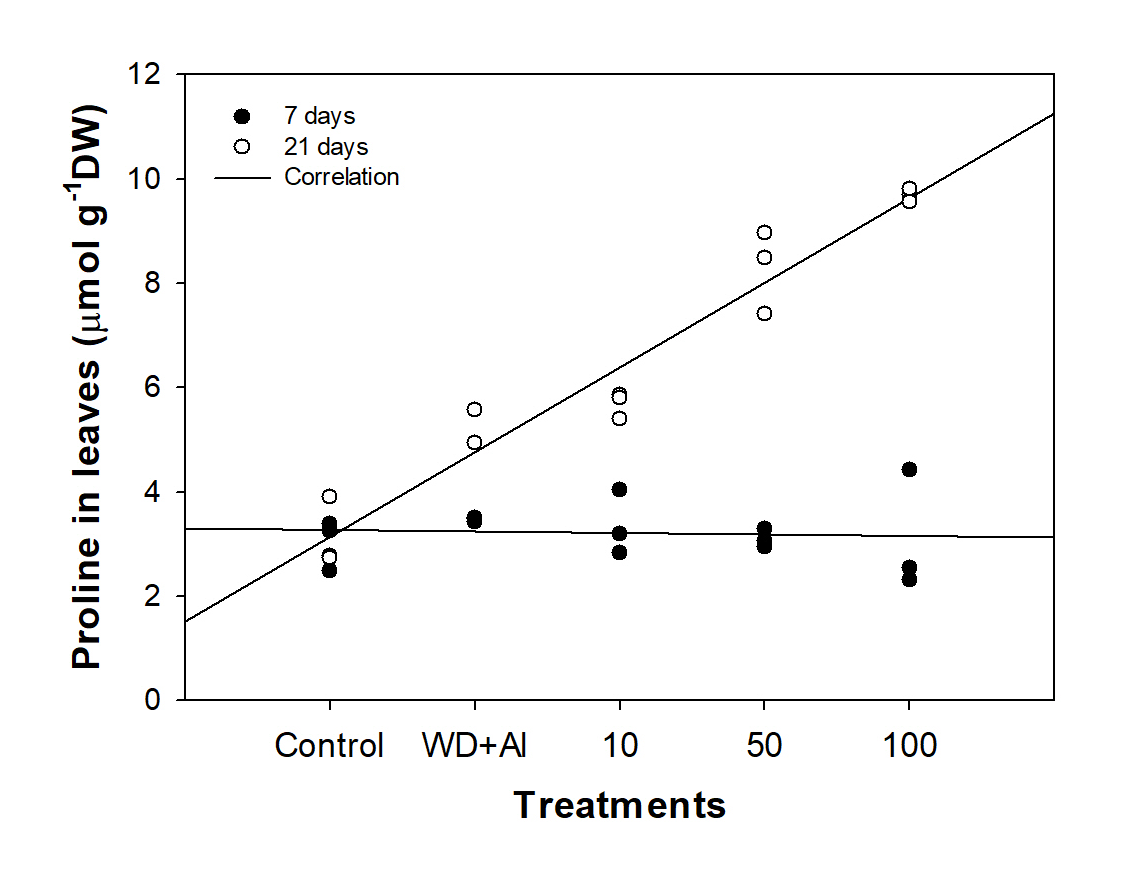

Supplement: Supplementary file 1 [file plants-14-03202-s001.zip › FigureS1.tiff]
